# Towards a unified approach for multiple myeloma care in Kenya - proceedings of the Inaugural Multiple Myeloma Congress

**DOI:** 10.11604/pamj.2021.40.236.30742

**Published:** 2021-12-17

**Authors:** Teresa Cherop Lotodo, Beatrice Jepngetich Melly, Kelvin Mogesa Manyega, Mercy Oduor, Valerie Magutu, Fredrick Chite Asirwa, Riyat Malkit, Alfred Karagu, Simon Onsongo, Caroline Wafula, Roselyne Yatich, Pravas Chandra Mishra, Austin Omondi, Diana Flora Namaemba, Yvette Oyollo, Maureen Kugo

**Affiliations:** 1 School of Medicine, Moi University, Eldoret, Kenya,; 2 Academic Model Providing Access To Healthcare (AMPATH) Oncology Institute, Eldoret, Kenya,; 3 Moi Teaching and Referral Hospital, Eldoret, Kenya,; 4 School of Pharmacy, Kabarak University, Nakuru, Kenya,; 5 Department of Human Pathology, University of Nairobi, Nairobi, Kenya,; 6 International Cancer Institute, Eldoret, Kenya,; 7 Aga Khan University Hospital, Nairobi, Kenya,; 8 National Cancer Institute, Kenya,; 9 School of Medicine, Maseno University, Kisumu, Kenya,; 10 Kakamega County Referral Hospital, Kakamega, Kenya,; 11 Max Super Specialty Hospital, New Delhi, India

**Keywords:** Multiple myeloma, medical education, stakeholder participation, Kenya

## Abstract

The rising burden of multiple myeloma in Kenya has not been met by a commensurate effort for control. Patients and practitioners struggle with unavailability and unaffordability of diagnostics, drugs and stem cell transplant leading to presentation at advanced stages and under-treatment with increased morbidities and mortality. A concerted effort among stakeholders is urgently needed to develop strategies for myeloma control. The scarcity of providers also carries grave consequences for Kenyan patients. The Academic Model Providing Access To Healthcare (AMPATH) multiple myeloma program organized the Inaugural Virtual Multiple Myeloma Congress to achieve both interactive specialist instruction and stakeholder engagement. Expert presenters and panellists from diverse disciplines were invited to offer in-depth presentations on myeloma care and case studies from panellists´ practice were used to contextualize learning points and form a basis for generating debate on the challenges facing providers and opportunities for care improvement. An audience of health professionals offering care to myeloma patients was invited. The underlying principle of recommendations developed during the congress was collaboration among in-country and international practitioners, researchers and policy experts from private and public sector. This partnership of stakeholders bears the potential of pooling scarce resources and for collective advocacy towards better patient care

## Conference proceedings

**Introduction:** an increasing number of new multiple myeloma (MM) cases has been documented. In the Eastern Africa region 2,530 new MM cases were reported for the year 2018 up from 1,900 in 2012 [1,2]. This is occurring in the background of lean access to diagnostic and treatment infrastructure. In Kenya, oncology services are concentrated in few urban centres forcing patients to travel long distances to access care which results in delayed diagnosis and impaired adherence to treatment [3]. Indeed, late presentation has been shown to contribute to poor survival of MM patients in sub-Saharan Africa settings [4].

The high cost of antimyeloma drugs remain a barrier to treatment access in low- and middle-income countries (LMICs) [5,6]. Additionally, Autologous Stem Cell Transplant (ASCT), which is currently considered standard treatment, is not available in most of sub-Saharan Africa including Kenya [7]. The scarcity of specialized personnel to diagnose and treat cancer in Kenya [8] is a problem that deserves special mention. Steps have been taken to improve MM care in Kenya including the introduction of an oncology cover under the National Hospital Insurance Fund (NHIF) which is a public insurer. National guidelines have also been developed [9] although concerns have been raised about successful guideline implementation [3], likely due to physical and human resource limitations. Initiatives to improve training of oncology personnel have also been reported [8]. In recent years the Academic Model Providing Access To Healthcare (AMPATH) MM program has conducted MM-specific training and awareness creation for healthcare professionals further boosting capacity to diagnose, refer and provide treatment across Kenya [10]. Nonetheless, the skill deficit required to provide universal and comprehensive access to MM care in Kenya is far from being filled. Regular symposia have been recommended as a mechanism for engaging stakeholders for the building of successful cancer control programs in LMICs [11]. Through such meetings, trends for research and practice are highlighted and issues that call for discussion are identified [12].

The Inaugural Virtual Multiple Myeloma Congress under the rallying call - 'towards a unified approach for MM care in Kenya' brought together leading clinical practitioners, researchers and policy experts with a multidisciplinary audience (Table 1, Table 2) to share expert instruction in an interactive format through presentations and case-based discussions. Moreover, participants shared their experiences, identified challenges and goals that need collective action. This article features key learning points that engendered debate and subsequent recommendations.

**Table 1:** participants of the Inaugural Virtual Multiple Myeloma Congress 2020

Professional category	Frequency	Percent
Medical officer/registrar/resident/physician	34	25.2
Pharmacist/oncology pharmacist/clinical pharmacist	20	14.8
Nurse/clinical research nurse/oncology nurse	16	11.9
Clinical officer/clinical officer-oncology	14	10.4
Lecturers	10	7.4
General pathologist/clinical pathologist	9	6.7
Administrators (ministry of health, non-government, hospital)	8	5.9
Oncologists (medical/radiation)	8	5.9
Haematologists	6	4.4
Others (social workers, biostatisticians, laboratory scientists, health records and information managers)	6	4.4
Pharmaceutical technologist	4	3.0
Total	135	100.0

**Table 2:** institutions represented at the Inaugural Virtual Multiple Myeloma Congress 2020

Institution type	Sectorial affiliation	Institution name
**Myeloma care**	**Public, Kenya**	
		Moi Teaching and Referral Hospital
		Kenyatta National Hospital
		Kenyatta University Teaching and Referral Hospital
		Jaramogi Oginga Odinga Teaching and Referral Hospital
		Coast General Hospital
		Nakuru County Referral Hospital
		Kakamega County Referral Hospital
		Nyeri County Referral Hospital
		Embu Level 5 Hospital
		Longisa County Referral Hospital
		Migori County Referral Hospital
		Kapsabet County Referral Hospital
		Kitale County Hospital
	**Faith-based, Kenya**	
		The Mater Hospital
		Tenwek Mission Hospital
		AIC Kijabe Mission Hospital
	**Private, Kenya**	
		Nairobi Hospital
		Aga Khan University Hospital
		MP Shah Hospital
		Metropolitan Hospital, Nairobi
	**International**	
		Max Super Specialty Hospital, New Delhi, India
	**Commercial laboratory, Kenya**	
		ScanLab
	**Pharmaceutical supplies, Kenya**	
		Medipoint EA LTD
		Philips Pharmaceuticals
**Academia**	**Kenya**	
		Moi University
		University of Nairobi
		Aga Khan University
		Maseno University
		Kenyatta University
		University of Kabianga
	**International**	
		Indiana University
		University of Zimbabwe
		Alexandria University
**Government**	**Policy**	
		National Cancer Institute (NCI)-Kenya
**Non-governmental**	**Care and research**	
		AMPATH
		International Cancer Institute (ICI), Eldoret
	**Professional**	
		Kenya Clinical Officers Association (KCOA)

AIC: Africa Inland Church, AMPATH: Academic Model Providing Access to Healthcare

### Day 1 plenary

**Moderator:** Dr. Teresa Lotodo (general pathologist and lecturer, Department of Pathology, Moi University).

**Panellist:** Dr. Simon Onsongo (clinical pathologist, lecturer, Maseno University).

**Theme 1 - multiple myeloma diagnosis in Kenya - challenges and opportunities:** after the opening address given by Dr. Jesse Opakas (senior medical/radiation oncologist, director, haematology/oncology, MTRH) a presentation was made by Dr. Valerie Magutu (clinical pathologist, lecturer, Hematology and Blood Transfusion, Department of Human Pathology, University of Nairobi). Her presentation centred on the pathologic diagnostic process for MM. Accessibility to MM diagnostics was a pervasive theme in the presentation, panel discussion and follow-up consultations. Accessibility to diagnostics for MM in Kenya is low owing to financial and geographic barriers. Tests required to diagnose and stage MM: serum protein electrophoresis, serum free light chain (FLC), immunophenotyping, β_2_ microglobulin and cytogenetics are not readily obtainable at both the basic and advanced levels of the public healthcare referral system, predominantly used by underserved populations, and may contribute to late diagnosis. In contrast, the private sector is capable of providing these services. A public-private-partnership has the potential of increasing access to underserved populations in the short-term. In Guatemala, a lower-middle income country with disparate access to cancer care, integration between a public paediatric cancer centre and private sector ensured that advanced diagnostic services are made available to vulnerable populations [11].

In the long-term, strategically located public healthcare facilities should be equipped with basic diagnostic infrastructure that will enable clinicians to establish prompt diagnosis and offer timely referrals if advanced care is needed. Not only will this reduce costs but it will also cut travelling costs which have been noted to impede care delivery [3]. This is typified by the efforts of non-profit organizations such as the Academic Model providing Access To Healthcare (AMPATH) MM program in Kenya which donates consumables (biopsy needles) and offers diagnostic training to healthcare professionals from peripheral healthcare facilities; or the acquisition of fixed equipment (digital mammography machines and video colposcopies) by the Partners for Cancer Care and Prevention (PFCCAP) in Colombia to improve breast and cervical cancer diagnosis rates [13]. However, to attain financial sustainability, the Kenyan government should increase its annual expenditure on cancer care to facilitate diagnostic infrastructural development [3,11]. Annex 1 is a transcript of the case-based panel discussion (pathologic diagnosis of MM).

**Screening:** the role of screening, which presents an opportunity for early diagnosis, was also a prominent sub-theme. In Kenya most MM diagnoses are made upon clinical manifestation which tends to be late and could be part of the reason why survival times are shorter. This makes the prospect of screening attractive in this setting. Findings from a population-based study show that MM patients with prior incidental diagnosis and follow-up of monoclonal gammopathy of undetermined significance (MGUS), a pre-myeloma condition, were found to have longer survival than patients in whom a diagnosis was made due to overt disease [14]. Current guideline recommendations for diagnosis, risk assessment for progression and follow-up of pre-myeloma states require tests including serum FLC, type and amount of serum M protein [15]. This further magnifies the need to improve diagnostic infrastructure for the Kenyan public health system. A trial (iStopMM) is underway to determine not only the survival benefits of screening and follow-up of MGUS but also quality of life, mental health and cost-effectiveness. Results of this trial may contribute to forming a stronger foundation to advocate for expansion of screening, already made available by NHIF for cervical and prostate cancers, to MM.

**Theme 2 - multiple myeloma research in Kenya:** Dr. Beatrice Melly (clinical haematologist, Moi Teaching and Referral Hospital) delivered a presentation on the status of MM research in Kenya. Highlights included the unexplored research landscape in the country and the peculiarities of MM in African patients. Myeloma research in Kenya is scanty. Reports have mainly focused on clinical presentation, treatment and survival [16,17], a picture that also applies to other African countries [4,18]. There are no open myeloma clinical trials in Africa currently. Kenya however, shows great promise for innovative oncology research as it ranks fourth on the list of African countries with the highest number of open oncology clinical trials [19].

Multiple myeloma displays marked differences in biology, epidemiology and response to therapy in blacks when compared with other racial groups. The prevalence of MGUS has been shown to be twice as high in Ghanaian blacks as compared to a white control group from the United States [20]. Multiple myeloma incidence was two, to three-fold higher and had an earlier age of onset in African Americans than in White Americans in a population-based study [21]. Furthermore, black race (as compared to whites) was identified as a risk factor for transformation of MGUS to MM (hazard ratio 1.98) [22]. On the positive side, when offered the same standard-of-care treatment, African Americans have been shown to have a higher myeloma-specific survival than whites [23,24]. It is thought that the above differences are mediated by differences at the biological level. In fact, the favourable survival outcomes among blacks are thought to be due to lower frequencies of high risk cytogenetic abnormalities such as t(4;14) and del(17p) [25,26], and excess prevalence of the hyperdiploid karyotype [27,28] as compared to whites.

Given the unique characteristics of MM in Africans, research efforts specially focused on local African populations are required to optimize prevention, diagnosis and treatment interventions [29]. In Kenya, such research should include studies on prevalence of pre-malignant conditions (MGUS, smouldering MM), frequency of biomarkers including cytogenetic and genomic abnormalities and mechanistic studies to determine the role of genomic biomarkers in progression to MM from pre-malignant states. This research has the potential for devising techniques to predict progression and identifying potential therapeutic targets that may aid in interception of early disease. The value of operational research, to evaluate impact of current interventions on patient outcomes, cannot be overstated. Outcomes may be clinical such as response rates, survival rates, adverse event rates; humanistic - health-related quality of life; or economic - cost-of-illness, cost effectiveness e.t.c. Importantly, reporting on ingenious models for care delivery will empower stakeholders to overcome resource scarcity [11], a major barrier to care delivery in Kenya [3].

**Myeloma registration:** attention was drawn to MM registration and its potential value for MM research and control. High quality population-based cancer registration (PBCR) in Kenya is in its nascent stages. The Nairobi Cancer Registry and the Eldoret Cancer Registry, both representing subnational populations, are the only PBCRs that contributed data to ‘cancer in sub-Saharan Africa (2018)´ - a publication of cancer incidence from the International Agency for Research on Cancer [30]. Both registries are members of the African Cancer Registry Network (AFCRN). While the current focus of Kenyan PBCRs is on incidence and mortality data for the broader public health agenda of cancer control one cannot ignore the need for site-specific cancer data. The Swedish Myeloma Registry is an example of a PBCR with not only high completeness and accuracy but also myeloma-specific data including pertinent diagnostic and treatment particulars: cytogenetics, M-protein isotype, bone marrow plasma cell percentage (BMPC), serum FLC, CRAB criteria (CRAB: calcium, renal insufficiency, anemia or bone lesions); first-line therapy, occurrence and date of first relapse or complications [31]. In the face of resource limitations in Kenya, establishing a similar PBCR may not be achievable in the near future. However, hospital-based registration and aggregation at national level of clinical MM data is a feasible alternative that may provide crucial insight into the effectiveness of current clinical interventions in various cancer centres including disparities in care delivery. In Australia, a similar approach has been used to generate useful treatment and outcome estimates for vulval cancer, a rare cancer, for which PBCRs lacked stage, grade and treatment data [32]. With the scarcity of trials in Kenya observational registry research may provide an important data source to shape evidence based policy formulation and clinical practice.

**Myeloma working group:** collaboration among researchers, healthcare workforce and policy makers was suggested as one way to generate creative solutions for the complex problem of delivering high quality research and care in the background of increasing MM cases and stretched resources. Translating research findings to practice also requires close collaboration between researchers and practitioners [33]. In research, collaboration is often fraught with inadequate funding, competing interests for personal time among team members, difference in research infrastructure of collaborating institutions, and administrative workload in obtaining approvals for a single protocol in different sites [34]. The difference between the rigorous scientific approach of researchers and the quick decision process employed by practitioners also presents a problem during collaborative work [35]. On the other hand, facilitators for collaboration include the desire for contribution to practice-changing research, authorship recognition and belonging to a community of researchers [34]. The primacy of skilled project management and human factors such as leadership, shared vision and proper communication have been emphasized in developing and sustaining collaborations in health research [35].

Collaboration may provide an avenue to conduct large prospective multi-centre studies [34] on MM in Kenya thus improving the quality and generalizability of findings. In addition, member institutions in a collaborative may benefit from sharing physical, human and intellectual resources (best practices). Greater visibility for MM through advocacy in research, policy and fundraising [36] can also be achieved using a collective approach.

### Day 2 plenary

**Moderator:** Dr. Beatrice Melly (clinical haematologist, MTRH).

**Panellists:** Dr. Caroline Wafula (clinical pharmacist, oncology, Jaramogi Oginga Odinga Teaching and Referral Hospital [JOOTRH]), Dr. Pravas Chandra Mishra (director - Haematology & BMT Max Super Specialty Hospital, Patraparganj & Vaishali, New Delhi India), Ms. Roselyne Yatich (senior oncology nurse, MTRH).

**Theme 3 - treatment and laboratory monitoring for multiple myeloma in Kenya:** a presentation was given by professor Malkit Riyat (consultant haematologist, Aga Khan University Hospital, (AKUH)) on treatment and laboratory monitoring. He recommended bortezomib-based three-drug induction regimens for Newly Diagnosed Multiple Myeloma (NDMM) with the aim of attaining the longest duration of response followed by maintenance therapy using lenalidomide. Both of these approaches have been documented to have significant survival advantages [37,38]. However, the high costs of these drugs cannot be overlooked.

The high cost of MM therapeutics is a major setback to optimal patient outcomes in Kenya. Patented cancer drugs are less affordable in low and middle income countries (LMICs) as compared to high-income countries; the difference in affordability being driven mainly by the low incomes in LMICs [5]. Despite the underlying economic forces driving this disparity, ethical questions are abound as to whether the current situation where poor global communities are unable to access effective cancer treatment should persist. Advocates are calling for differential pricing in LMICs, especially for drugs with substantial clinical benefits [5], in the case for MM - bortezomib and lenalidomide. Pharmaceutical companies can also mitigate high drug costs by introducing patient assistance programs. Takeda´s NINLARO® (ixazomib) patient assistance program for relapsed MM is one such example.

**Theme 4 - stem cell transplant, MM treatment advances & opportunities in Kenya:** in his address, professor Fredrick Chite (consultant physician, medical oncologist/haematologist, director, International Cancer Institute (ICI)) touched on, among other topics, transplant eligibility, timing of transplant, stem cell mobilization and transplantation, transplant complications, engraftment and discharge and post-transplant care. Worth noting is the centrality of bone marrow transplant in therapy for MM. In the *Intergroupe Francophone du Myélome (IFM)* 2009 trial, patients receiving transplant with lenalidomide/bortezomib/dexamethasone (RVd) reported higher median progression free survival - PFS (50 vs 36 months respectively) than patients receiving RVd alone. Benefits in complete response (CR) and minimal residual disease (MRD) negativity rate were also recorded [39]. Recent evidence from the GRIFFIN trial appears to further cement the place of transplant in MM therapy. Patients who received a four drug induction regimen of daratumumab + RVD achieved a deeper response after transplant with more patients achieving stringent complete response and very good partial response [40].

Unfortunately, in Kenya only few patients who can afford to travel to India or other countries with bone-marrow transplant infrastructure can access this service. The Aga Khan University Hospital, a private tertiary hospital, plans to set up the first bone-marrow transplant service in the country. Participants re-expressed sentiments on public-private partnerships as a mechanism to provide access to underserved communities in Kenya when the transplant service finally becomes operational. In the absence of bone-marrow transplant in the country a focus on acquisition of new highly effective therapies that confer superior survival outcomes is an appealing stop-gap measure. Daratumumab for instance when added to bortezomib/melphalan/prednisone (VMP) results in higher 3-year overall survival (78% vs 68%) as compared to VMP alone in transplant ineligible patients not to mention benefits in PFS [41]. Carfilzomib based therapies, on the other hand, are improving outcomes for relapsed/refractory MM [42] whereas chimeric antigen receptor (CAR) T cell therapies [43], still under development, show promise in heavily pre-treated patients. Access to these new agents for Kenyan patients calls for participation in international multi-centre randomized studies. Annex 2 is a transcript of the case-based panel discussion under treatment, monitoring and stem cell transplant.

**Improving access to myeloma care - role of government:** Dr. Alfred Karagu (chief executive officer, National Cancer Institute of Kenya (NCI-K)) offered an overview of NCI-K, its history and core mandate which involves coordination and overseeing cancer control activities at the national level under the Ministry of Health (MoH). Issues of interest to MM care that are under the purview of NCI-K include access to care, infrastructural and human resource capacity building, surveillance, research and quality of cancer care. In response to the problem of unavailability of diagnostic services for MM Dr. Karagu noted that the government´s ongoing response is the decentralization of cancer control services to county health facilities in line with the National Cancer Control Strategy [44]. This together with capacity building for infrastructure and personnel will bring services closer to patients thus reducing delays in diagnosis. On the aspect of unaffordability of MM treatment he acknowledged challenges arising from the coverage limits imposed by NHIF on cancer treatment. Among MM patients, treatment interruption due to depleted cover and delays to treatment initiation occasioned by long waits before new subscribers can access benefits are pressing problems. The push for comprehensive coverage of cancer care by NHIF is a debate in progress that will hopefully yield fruits.

The Government of Kenya through MoH and NCI-K is the most important stakeholder in the cancer control sector according to a stakeholder analysis by Makau-Barasa *et al*. (2020), considering its role in developing policy and allocation of resources, human and financial, for policy implementation [45]. This justifies its involvement in efforts to improve MM care in Kenya.

**Recommendations:** several recommendations arose from the congress. Key among these was the need for collaborations between practitioners and researchers both in private and public sector. Such a partnership provides the possibility of sharing of scarce diagnostic and treatment infrastructure. It also provides the opportunity for carrying out more impactful observational research. Secondly, acquisition of diagnostic equipment for public health facilities and the guarantee of comprehensive public insurance for MM care are necessary steps that need to be undertaken by the government. The pharmaceutical industry may also contribute to affordability of care by implementing differential pricing and/or patient assistance programs. Table 3 is a summary of recommendations arising from the congress. The congress concluded after the closing remarks from Mercy Oduor (program coordinator, AMPATH multiple myeloma).

**Table 3:** recommendations for improving myeloma care in Kenya

1	Engender public-private partnerships as a means to providing access to advanced diagnostics and therapies (e.g. cytogenetics and stem cell transplant) to underserved communities
2	Government to facilitate infrastructure upgrade in selected tertiary and peripheral facilities of basic MM diagnostics such as SPEP, FLC and β_2_ microglobulin
3	Collaboration with international researchers with capacity for screening, MM biology and therapeutic trials
4	Focus on local operational research on clinical, HRQoL, economic outcomes and creative service delivery models
5	Aggregate clinical data form hospital-based MM registries for greater quality research
6	Local collaboration for research, resource sharing and collective advocacy in policy and fundraising
7	Engage pharmaceutical companies for differential pricing of drugs and/or patient assistance programs to increase affordability
8	Advocate for comprehensive insurance plans that cover diagnostics and adequate treatment of MM
9	NGOs already conducting MM training to support government efforts for decentralization of oncology services through capacity building for MM care
10	Conduct recurring congresses to build momentum for greater collaboration and take account of progress towards common goals

MM: multiple myeloma; SPEP: serum protein electrophoresis; FLC: free light chain; HRQoL: health related quality of life; NGOs: non-governmental organizations

## Annexes

Annex 1: a transcript of the case-based panel discussion under pathologic diagnosis of MM (PDF-100kb)

Annex 2: a transcript of the case-based panel discussion under treatment, monitoring and stem cell transplant (PDF-103kb)
